# Epitope analysis of anti-myeloperoxidase antibodies in propylthiouracil-induced antineutrophil cytoplasmic antibody-associated vasculitis

**DOI:** 10.1186/ar4386

**Published:** 2013-11-20

**Authors:** Chen Wang, Shen-ju Gou, Peng-cheng Xu, Ming-hui Zhao, Min Chen

**Affiliations:** 1 Renal Division, Department of Medicine, Peking University First Hospital, Beijing 100034, China; 2 Peking University Institute of Nephrology, Beijing 100034, China; 3 Key Laboratory of Renal Disease, Ministry of Health of China, Beijing 100034, China; 4 Key Laboratory of Chronic Kidney Disease Prevention and Treatment (Peking University), Ministry of Education, No 8, Xishiku Street, Xicheng District, Beijing 100034, China

## Abstract

**Introduction:**

Increasing evidence has suggested that linear epitopes of antineutrophil cytoplasmic antibody (ANCA) directed to myeloperoxidase (MPO) might provide clues to the pathogenesis of propylthiouracil (PTU)-induced ANCA-associated vasculitis (AAV). This study mapped epitopes of MPO-ANCA in sera from patients with PTU-induced MPO-ANCA (with or without vasculitis) and primary AAV, aiming to analyze certain epitopes associated with the development of PTU-induced AAV.

**Methods:**

Six recombinant linear fragments, covering the whole amino acid sequence of a single chain of MPO, were produced from *Escherichia coli*. Sera from 17 patients with PTU-induced AAV, 17 patients with PTU-induced MPO-ANCA but without clinical evidence of vasculitis, and 64 patients with primary AAV were collected at presentation. Of the 17 patients with PTU-induced AAV, 12 also had sera at remission. The epitope specificities were detected by enzyme-linked immunosorbent assay by using the recombinant fragments as solid-phase ligands.

**Results:**

Compared with patients with PTU-induced MPO-ANCA but without clinical vasculitis, sera from PTU-induced AAV patients showed significantly higher reactivity against the H1 fragment of MPO (optical density values: 0.17 (0.10 to 0.35) versus 0.10 (0.04 to 0.21), *P* = 0.038) and could recognize a significantly higher number of fragments (two (none to four) versus one (none to two), *P* = 0.026). Compared with sera from primary AAV patients, sera from PTU-induced AAV patients had significantly higher reactivity to the P fragment and the H4 fragment (47.1% versus 14.1% *P* < 0.001; 41.2% versus 14.1%, *P* = 0.034, respectively), and could recognize a significantly higher number of fragments (two (none to four) versus one (none to two), *P* = 0.013]. Among the 12 PTU-induced AAV patients with sequential samples, the number of fragments recognized in remission was significantly less than that in initial onset (two (none to four) versus none (none to 0.75), *P* < 0.001].

**Conclusions:**

Linear epitopes of MPO molecules might be associated closely with PTU-induced AAV. In particular, the P and H4 fragments may be important epitopes in PTU-induced AAV.

## Introduction

Antineutrophil cytoplasmic antibody (ANCA)-associated vasculitis (AAV) includes granulomatosis with polyangiitis (GPA), microscopic polyangiitis (MPA), and eosinophilic granulomatosis with polyangiitis (EGPA). ANCAs are serologic hallmarks for the previously mentioned primary small-vessel vasculitis. Proteinase 3 (PR3) and myeloperoxidase (MPO) are the two most important target antigens of ANCA [1,2]. ANCAs are also involved in the pathogenesis of AAV [3-5]. One of the most important developments in the ANCA field is the increasing recognition of a number of drugs that could induce AAV. Among these drugs, the most often implicated one is propylthiouracil (PTU) [6,7], a common antithyroid agent. It was reported that most patients with PTU-induced AAV are MPO-ANCA positive [8]. The immunologic characteristics of MPO-ANCA, including IgG subclasses, titers, avidity, and epitopes, are contributors to PTU-induced AAV [9-13].

Many similarities in clinical manifestations are present between PTU-induced AAV and primary AAV. However, it has been suggested that the mechanism involved in the synthesis of PTU-induced MPO-ANCA might be different from that in patients with primary AAV [12]. For example, our previous study preliminarily suggested that epitopes recognized by PTU-induced MPO-ANCA were different from those recognized by MPO-ANCA from patients with primary AAV [12]. By investigating the association between epitope profiles and clinical manifestations of PTU-induced AAV in children, Fujieda *et al.*[13] speculated that the clonality of MPO-ANCA might be a risk factor for developing vasculitis. These findings implied that epitope mapping of MPO, especially linear epitopes, might draw some distinction between PTU-induced AAV and primary AAV, and provide some clues for exploring the pathogenesis of PTU-induced AAV.

In patients with PTU-induced AAV, after discontinuation of the offending drug and initiation of immunosuppressive treatment, patients often achieve remission quickly, with ANCA avidity and titers declining [11]. Investigating epitopes of MPO in sequential serum samples (that is, in active stage and remission of PTU-induced AAV) may help us to find the epitope(s) associated with active diseases.

Moreover, in our previous cross-sectional study, it was found that among patients with PTU-induced ANCA, only about one in five developed clinically evident vasculitis [14]; even in those who were MPO-ANCA positive, not all developed clinically evident vasculitis [15]. It is reasonable to investigate whether the difference of epitope(s) of MPO contribute to the development of these two different phenotypes (that is, with or without vasculitis).

With these previously mentioned questions in mind, we produced six linear recombinant deletion mutants of the MPO molecule and mapped the epitopes of MPO-ANCA in sera from both patients with PTU-induced MPO-ANCA (with or without vasculitis) and patients with primary MPO-AAV.

## Methods

### Patients and sera

Seventeen patients with PTU-induced AAV, diagnosed at Peking University First Hospital from October 1999 to October 2005, were recruited in this study. All the patients met the criteria of the Chapel Hill Consensus Conference definition of AAV [16]. PTU-induced AAV was defined as follows: (a) the signs and symptoms of vasculitis were temporally related to using PTU, and regressed with its discontinuation; (b) serum ANCA was positive, especially in those with multi-antigenicity; and (c) medical conditions that mimicked vasculitis were excluded, especially infections and malignancies, and other definable types of vasculitis [17]. At the time of diagnosis, all the patients were positive for MPO-ANCA. Serum samples were collected from the 17 patients on diagnosis before the initiation of immunosuppressive treatments. Serum samples from 12 of these 17 patients who achieved remission were collected at their regular ambulatory visits. Serum MPO-ANCA in all these 12 patients remained positive despite clinical remission. Remission was defined as “absence of disease activity attributable to active disease qualified by the need for ongoing stable maintenance immunosuppressive therapy”, as described previously [18]. Serum samples were also collected from the following participants: (a) 17 patients with PTU-induced serum MPO-ANCA but without clinical evidence of vasculitis; (b) 64 patients with primary AAV that were MPO-ANCA positive; (c) three patients with PTU-induced lupus. PTU-induced lupus was defined as previously described [19]; and (d) 35 healthy blood donors were collected as normal controls. Sera from all subjects were obtained and kept at −70°C until use.

The research was in compliance of the Declaration of Helsinki and approved by the ethics committee of Peking University First Hospital. Written informed consent was obtained from each participant.

### Detection of MPO-ANCA

All sera were screened for ANCA with indirect immunofluorescence by using precooled ethanol-fixed normal peripheral neutrophils as substrate, according to the manufacturer (Euroimmun, Lübeck, Germany), and MPO-ANCAs were measured with enzyme-linked immunosorbent assay (ELISA), as described previously [20].

### Preparation of recombinant MPO fragments

Six recombinant linear fragments, covering the whole-length amino acid sequence of a single chain of MPO (that is, P, L, H1, H2, H3, and H4) were prepared as deletion mutants of MPO from *Escherichia coli*, as described in our previous study [21]. The amino acid sequences of the six fragments were as follows: 49 to 164 for propeptide (P), 165 to 272 for light chain (L), 279 to 409 for the N terminal of the heavy chain (H1), 399 to 519 for the second part of the heavy chain (H2); similarly, 510 to 631 for H3 and 622 to 745 for H4. All the six recombinant MPO fragments were highly purified as proteins tagged with histidines with >80% purity by Ni-NTA column chromatography (an additional figure shows this in more detail (see Additional file 1)). The mature MPO, which is produced by two heavy-light protomer units interacting, is a symmetric homodimer of approximately 150 kDa, with each half linked by a disulfide bond between C319 residues of the heavy subunit (additional figures show this in more detail [see Additional files 2 and 3].

### Determination of the reactivity of recombinant fragment of MPO by ELISA

Highly purified recombinant MPO fragment were reconstituted to 10 μg/ml with coating buffer (0.05 *M* bicarbonate buffer, pH 9.6). A 100-μl portion of the mixture was then plated to a well of a polystyrene microtiter plate (Nunc Immunoplate; Nunc, Roskilde, Denmark) and kept overnight at 4°C. Every plate contained native MPO (2 μg/well) as a positive antigen control. The plate was washed 3 times with PBS containing 0.1% Tween-20 (PBST) (Chemical Reagents, Beijing, China). Then 2% BSA diluted by PBS was used to block the nonspecific binding sites. The sera of subjects were diluted to 1:100 by PBST/0.5 *M* NaCl (NaCl 0.5 *M*, KCl 2.7 m*M*, Na_2_HPO_4_ 10 m*M*, KH_2_PO_4_ 2 m*M*, pH 7.4), and were added in duplication. Every plate contained positive, negative, and blank controls. The plate was incubated at 37°C for 1 hour and then washed 3 times with PBST, and the binding was detected with alkaline phosphatase-conjugated goat anti-human IgG (Fc specific; Sigma, St. Louis, MO, USA) at a dilution of 1:5,000. The plate was washed 3 times with washing buffer and the P-nitrophenyl phosphate (pNPP, 1 mg/ml; Sigma) was used in substrate buffer [1 *M* diethanolamine and 0.5 m*M* MgCl_2_ (pH 9.8)]. The results were recorded as the absorbance at 405 nm (A 405 nm), and samples were considered positive if the A 405 nm exceeded mean + 2 SD of the A 405 nm of the sera from 35 normal blood donors.

### Detection of anti-endothelial cell antibodies and autoantibodies directed to specific ANCA antigens other than MPO

AECA as well as ANCA directed to six specific target antigens, including proteinase 3, cathepsin G, lactoferrin, human leukocyte elastase (HLE), azurocidin, and bactericidal/permeability-increasing protein (BPI) were examined. AECA was detected by Western blot analysis, and ANCA directed to the previously mentioned six specific target antigens were detected with ELISA, as described in our previous study [22,23].

### Statistical analysis

Differences of quantitative parameters between groups were assessed by using the *t* test (for data that were normally distributed) or nonparametric test (for data that were not normally distributed). Differences in qualitative data were compared by using χ^2^ tests. The difference was considered significant if a *P* value was <0.05. Analysis was performed with SPSS statistical software package (version 18.0, Chicago, IL, USA).

## Results

### General data of the patients

Among the 17 PTU-induced AAV patients, 15 were female and two were male patients, with an age of 30.8 ± 15.2 (range, 11–58) years at diagnosis. The Birmingham Vasculitis Activity Scores (BVASs) were 17.1 ± 5.5 (range, 7 to 31 years). The level of initial serum creatinine was 75.62 ± 40.64 μ*M*. Among the 64 primary AAV patients, 32 were male and 32 were female patients, with an age of 60.5 ± 15.1 (range, 15 to 83) years at diagnosis. The BVASs were 20.27 ± 5.18 years (range, 13 to 36 years) (Table 1).

**Table 1:** Clinical data of 17 patients with PTU-induced AAV and 64 patients with primary AAV

	**PTU-induced AAV**	**Primary AAV**
	**(*n*** **= 17)**	**(*n*** **= 64)**
Male/female	2/15	32/32
Age (years)	30.8 ± 15.2	60.48 ± 15.14
Scr (μ*M*)		
Mean ± SD	75.62 ± 40.64	339.11 ± 237.71
Range	38-176.9	70-1007
Renal insufficiency at diagnosis	13 (76.4%)	48 (75%)
ESR (mm/1 hour)	46.92 ± 38.86	70.05 ± 40.14
Skin rash	5(29.4%)	7 (10.9%)
Arthralgia	9(52.9%)	15(23.4%)
Muscle pain	6 (35.3%)	10 (15.6%)
Lung	6 (35.3%)	43 (67.2%)
ENT	6 (35.2%)	27(42.2%)
Ophthalmic	2 (11.8%)	14(21.9%)
Gastrointestinal	3(17.6%)	11(17.2%)
Nervous system	1 (5.9%)	10(15.6%)
BVAS		
Mean ± SD	17.1 ± 5.5	20.27 ± 5.18
Range	7-31	13-36

AAV ANCA-associated vasculitis, BVAS Birmingham Vasculitis Activity Scores, ENT ear, nose, and throat, SD standard deviation.

Among the 12 PTU-induced AAV patients at remission, 10 were female and two were male patients, with an age of 34.7 ± 20.0 (range, 11 to 76) years at diagnosis. The BVAS levels were all zero (Table 2).

**Table 2:** Cutoff values in the assay of autoantibodies against various linear fragments of MPO

**Epitope**	**Mean**	**SD**	**Cutoff values**
Pre	0.058	0.033	0.124
H1	0.076	0.066	0.208
H2	0.074	0.049	0.172
H3	0.061	0.040	0.141
H4	0.094	0.070	0.234
L	0.051	0.043	0.137

Among the patients with PTU-induced MPO-ANCA, except for only one with clinical vasculitis had positive ANA, all the patients with and without clinical vasculitis were negative for ANA, anti-dsDNA, anti-histone, and anti-Sm antibodies.

Among the three patients with PTU-induced lupus, all were women, with age of 17, 30 and 57 years at diagnosis, respectively. All these three patients were positive serum ANA.

### The reactivity of MPO-ANCA against the linear fragments of MPO

**Figure 1:**
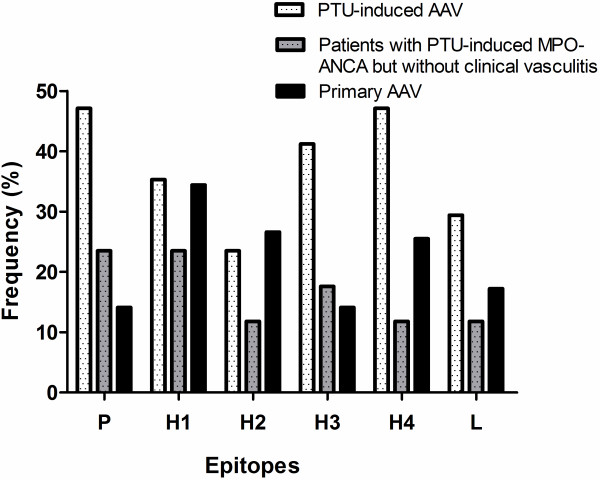
The frequencies of sera binding to different protein fragments of MPO in patients with PTU-induced AAV, patients with primary AAV, and patients with PTU-induced MPO-ANCA but without clinical vasculitis.

The cutoff values, which were set as the mean + 2 SD of A405 nm of the sera from 35 normal blood donors, in the assay of autoantibodies against various linear fragments of MPO were listed in Table 2. Serum samples from these 17 PTU-induced AAV patients in active stage, 12 PTU-induced AAV patients in remission, and 17 PTU-induced MPO-ANCA-positive patients but without clinical evidence of vasculitis were demonstrated to recognize all the six constructed linear protein fragments. Sera from 15 (88.2%) of 17 patients with PTU-induced AAV and 11 (61.1%) of 18 patients with positive PTU-induced MPO-ANCA but without clinical vasculitis could recognize at least one peptide. Meanwhile, among the 64 primary AAV patients, 37 (57.8%) of 64 patients showed a positive reaction to one or more linear fragments of the MPO chain. The reactivity distributed throughout the MPO molecule (Figure 1). None of sera from patients with PTU-induced lupus could recognize the recombinant fragments of MPO, except that only one could recognize L chain. In the patients with PTU-induced AAV, no significant association was found between levels of antibodies against various epitopes of MPO and clinical parameters, including serum creatinine, ESR, C-reactive protein, numbers with organ involvement and BVAS.

#### Differences of epitopes between patients with PTU-induced MPO-ANCA with and without clinical vasculitis

**Figure 2:**
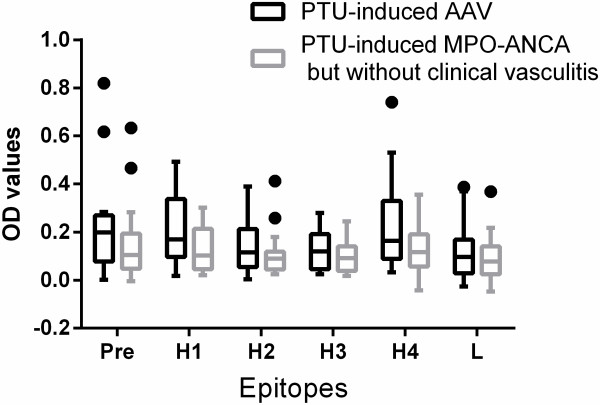
The OD values of sera binding to different protein fragments of MPO in patients with PTU-induced AAV and in patients with PTU-induced MPO-ANCA but without clinical vasculitis.

We compared the MPO epitopes between the 17 patients with PTU-induced AAV and the 17 patients with PTU-induced MPO-ANCA but without clinical vasculitis. Compared with sera from patients with PTU-induced MPO-ANCA but without clinical vasculitis, sera from PTU-induced AAV patients showed significantly higher reactivity against the H1 fragment of MPO [OD values: 0.17(0.10 to 0.35) versus 0.10(0.04 to 0.21), *P* = 0.038] (Figure 2). The number of fragments recognized in PTU-induced AAV was significantly more than that in PTU-induced MPO-ANCA but without clinical vasculitis (2(0 to 4) versus 1(0 to 2), *P* = 0.026).

#### Differences of epitopes between patients with PTU-induced AAV and with primary AAV

We compared the MPO epitopes between the 17 patients with PTU-induced AAV and the 64 patients with primary AAV. Compared with that from primary AAV patients, significantly higher proportions of sera from PTU-induced AAV patients had reactivity to P fragment and H4 fragment. (47.1% versus 14.1%; *P* < 0.001; 41.2% versus 14.1%; *P* = 0.034, respectively). The number of fragments recognized in sera from patients with PTU-induced AAV was significantly more than that in sera from patients with primary AAV (2(0 to 4) versus. 1(0 to 2); *P* = 0.013].

#### Differences of epitopes between patients with PTU-induced AAV in active stage and in remission

Among the 12 PTU-induced AAV patients with sequential samples (that is, in active stage and in remission), the number of fragments recognized in remission was significantly less than that in initial onset (2(0 to 4) versus 0(0 to 0.75); *P* < 0.001). Nine of the 12 patients at remission (patients 1, 2, 4, 5, 6, 7, 8, 9, and 11 in Table 3) recognized none of the six fragments, while the other 3 (patient No. 3, 10 and 12 in Table 3) recognized one or three fragments, respectively. The binding to linear fragments in remission was limited to the P, H1, H2, and H4 fragments. Among the four patients (patients 3, 5, 10, and 12 in Table 3) who had antibodies to H4 in the active stage, antibodies to H4 turned negative in two patients (patients 5 and 12 in Table 3) in remission; among the other eight patients who were seronegative for H4 during the active stage, none developed reactivity to H4 in remission.

**Table 3:** The reactivity of MPO-ANCA against the linear peptides of MPO of the 12 PTU-induced AAV patients with both onset and remission

**Patient number**	**Peptides recognized**	
	**Initial onset**	**Remission**
1	-	-
2	H1	-
3	H2/H4	P/H1/H4
4	H1/H3/L	-
5	H1/H2/H3/H4	-
6	H1/H3	-
7	L	-
8	P/H3	-
9	P	-
10	P/H1/H3/H4/L	H1/H4
11	P	-
12	P/H1/H2/H3/H4	H2

-, negative for any fragment test.

### The prevalence of AECA and autoantibodies directed to specific ANCA antigens other than MPO

Fifteen of 17 patients with PTU-induced vasculitis were serum AECA positive, whereas none of the patients with PTU-induced MPO-ANCA but without clinical vasculitis was AECA positive (88.2% versus None; *P* < 0.001). Meanwhile, the prevalences of antibodies against the proteinase 3, cathepsin G, lactoferrin, HLE, and azurocidin were significantly higher in patients with PTU-induced vasculitis than those in patients with PTU-induced MPO-ANCA but without clinical vasculitis (Table 4). No significant correlation was found between the previously mentioned autoantibodies (that is, AECA and autoantibodies directed to specific ANCA antigens other than MPO, and antibodies against various linear fragments of MPO.

**Table 4:** The prevalence of AECA and autoantibodies directed to specific antigens of ANCA

**AECA and ANCA directed to specific antigens other than MPO**	**Number and percentage with ANCA specificity**		
	**Patients with PTU-induced vasculitis**	**Patients with PTU-induced MPO-ANCA but without clinical vasculitis**	***P *** **value**
	**(*n*** **= 17)**	**(*n*** **= 11)**	
**AECA**	15 (88.2%)	0 (0)	<0.001
**PR3**	5 (29.4%)	1 (9.1%)	0.380
**HLE**	12 (70.6%)	3 (27.3%)	0.011
**Lactoferrin**	14 (82.4%)	5 (45.5%)	0.010
**Cathepsin G**	11 (64.7%)	0 (0)	<0.001
**BPI**	0 (0)	1 (9.1%)	0.209
**Azurocidin**	12 (70.6%)	1 (9.1%)	<0.001
**Number of ANCA target antigens recognized**	3 (2–4)	0 (0–2%)	<0.001

PR3, Proteinase 3; HLE*,* human leukocyte elastase; BPI, bactericidal/permeability-increasing protein.

## Discussion

Cumulative evidence has proved the pathogenic role of ANCA, in particular, MPO-ANCA, in the development of AAV. ANCA can mediate the activation of primed neutrophils, resulting in a respiratory burst and degranulation, which could play a direct pathogenic role in vasculitic lesions [3,24]. Xiao *et al.*[4] found that transfer of anti-MPO IgG from MPO-deficient mice immunized with mouse MPO into wild-type mice led to pauci-immune vasculitis. Our previous studies found that in PTU-induced AAV, the most important target antigen of ANCA is MPO [14], and the immunologic characteristics of MPO-ANCA are associated with the development of PTU-induced ANCA-associated vasculitis [25,26].

MPO epitopes recognized by human sera were both conformational and linear epitopes [27,28]. The current study investigated linear epitopes of MPO in patients with PTU-induced ANCA-associated vasculitis. Our previous study found that among patients with PTU-induced ANCA, the prevalence of serum MPO-ANCA was significantly higher in patients with clinical vasculitis than that in patients without clinical vasculitis [23]. Consistently, the current study found that sera from patients with PTU-induced AAV recognized significantly more fragments compared with sera from PTU-induced MPO-ANCA without clinical vasculitis. Furthermore, among the 12 PTU-induced AAV patients with sequential samples, the number of recognized epitopes declined rapidly once remission was achieved, whereas the levels of MPO-ANCA were persistently positive from active stage to remission. All these findings suggest that the linear epitopes, compared with conformational ones, might be associated more closely with PTU-induced AAV.

Compared with sera from primary AAV patients, sera from PTU-induced AAV patients could recognize significantly higher numbers of fragments, and had significantly higher reactivity to P fragment and H4 fragment. Moreover, among the four patients who had antibodies to H4 in the active stage, antibodies to H4 turned negative in two patients in remission; among the other eight patients who were seronegative for H4 during the active stage, no one developed reactivity to H4 in remission. These findings indicate that the linear epitope might be of more closely associated with PTU-induced AAV than that in primary AAV patients. However, the detailed role of antibody directed to the P and H4 fragment in the development of PTU-induced vasculitis demands further investigation.

We also found that PTU-induced AAV patients had higher reactivity against the H1 fragment compared with patients with PTU-induced MPO-ANCA but without clinical vasculitis. However, one patient with PTU-induced AAV was negative for H1 during the active stage but developed reactivity to H1 in remission. Therefore, the significance of the H1 fragment in PTU-induced AAV remains more uncertain.

Some limitations existed in our study. First, patients with PTU-induced AAV and patients with primary AAV were not age- or gender-matched because of the characteristics of these two diseases *per se*. Second, the sample size was relatively limited because PTU-induced AAV is an uncommon disease.

## Conclusions

The current study provided evidence that PTU-induced MPO-ANCA could recognize linear epitopes throughout the corresponding antigen molecule MPO. Linear epitopes of the MPO molecule, compared with conformational ones, might be associated more closely with PTU-induced AAV. In particular, the P and H4 fragments may be important epitopes in PTU-induced AAV.

## Abbreviations

AAV: ANCA-associated vasculitis; AECA: Anti-endothelial cell antibodies; ANCA: Antineutrophil cytoplasmic antibody; BPI: Bactericidal/permeability-increasing protein; BVAS: Birmingham Vasculitis Activity Score; EGPA: Eosinophilic granulomatosis with polyangiitis; GPA: Granulomatosis with polyangiitis; HLE: Human leukocyte elastase; MPA: Microscopic polyangiitis; MPO: Myeloperoxidase; PR3: proteinase 3; PTU: Propylthiouracil.

## Competing interests

The authors declare that they have no competing interests.

## Authors’ contributions

CW carried out the experiments, analyzed the data, and wrote and revised the manuscript. SJG and PCX participated in the design of the study and contributed reagents/materials/analysis tools. MHZ and MC designed, coordinated, and directed the study, and helped to write and revise the manuscript. All authors read and approved the final manuscript.

## Supplementary Material

Additional file 1: Figure S1 Schema of linear epitopes of MPO-ANCA. Description: Six recombinant linear fragments, P, L, H1, H2, H3, and H4, were produced by using *Escherichia coli*. P represents propeptide part, amino acids (aa) 49 to 164; L represents light chain, aa165 to 272; H1 to H4 represent four fragments of the heavy chain, H1 for aa279 to 409, H2 for aa399 to 519, H3 for aa510 to 631, and H4 for 622 to 745. About 10 amino acids overlapped between the two adjacent fragments on the heavy chain. Click here for file

Additional file 2: Figure S2 Structure of mature MPO. Description: The mature MPO is produced by two heavy-light protomer units interacting, which then formed a symmetric homodimer of approximately 150 kDa, with each half linked by a disulfide bond (MMDB ID 48480). Click here for file

Additional file 3: Figure S3 Location of L, H1, H2, H3, and H4 fragments on MPO. Description: L, H1, H2, H3, and H4 fragments were marked in different colors in one heavy-light protomer unit (MMDB ID 75307) as follows: L by purple; H1, H2, H3, and H4 by yellow. The second row was viewed from the opposite side of the first row. Click here for file
