# Supplementary figures and images for: Epitope analysis of anti-myeloperoxidase antibodies in propylthiouracil-induced antineutrophil cytoplasmic antibody-associated vasculitis
Source: Arthritis Res Ther. 2013 Nov 20;15(6):R196. doi: 10.1186/ar4386 (PMC3979166; doi:10.1186/ar4386)

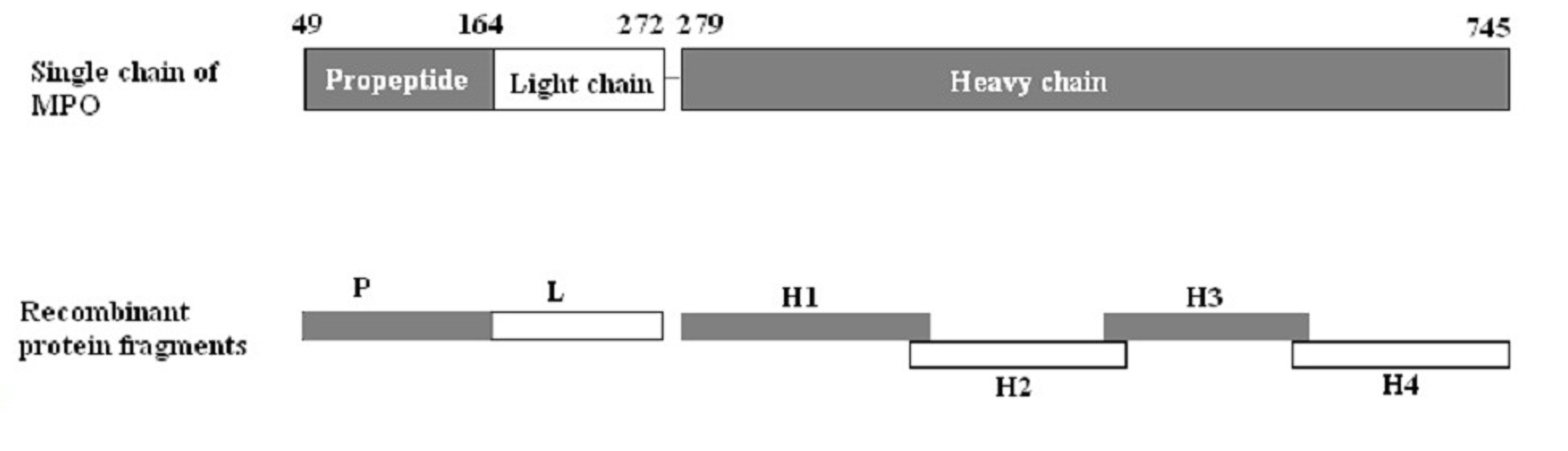

Supplement: Additional file 1: Figure S1 — Schema of linear epitopes of MPO-ANCA. Description: Six recombinant linear fragments, P, L, H1, H2, H3, and H4, were produced by using Escherichia coli. P represents propeptide part, amino acids (aa) 49 to 164; L represents light chain, aa165 to 272; H1 to H4 represent four fragments of the heavy chain, H1 for aa279 to 409, H2 for aa399 to 519, H3 for aa510 to 631, and H4 for 622 to 745. About 10 amino acids overlapped between the two adjacent fragments on the heavy chain. [file ar4386-S1.tiff]

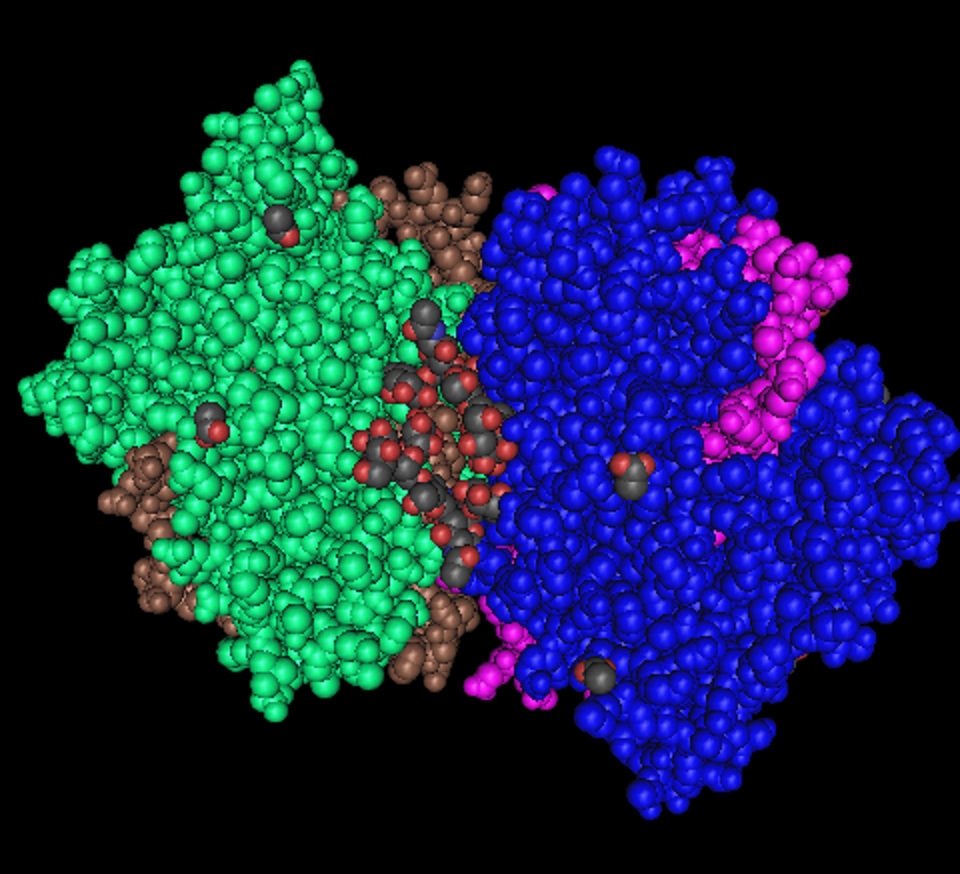

Supplement: Additional file 2: Figure S2 — Structure of mature MPO. Description: The mature MPO is produced by two heavy-light protomer units interacting, which then formed a symmetric homodimer of approximately 150 kDa, with each half linked by a disulfide bond (MMDB ID 48480). [file ar4386-S2.tiff]

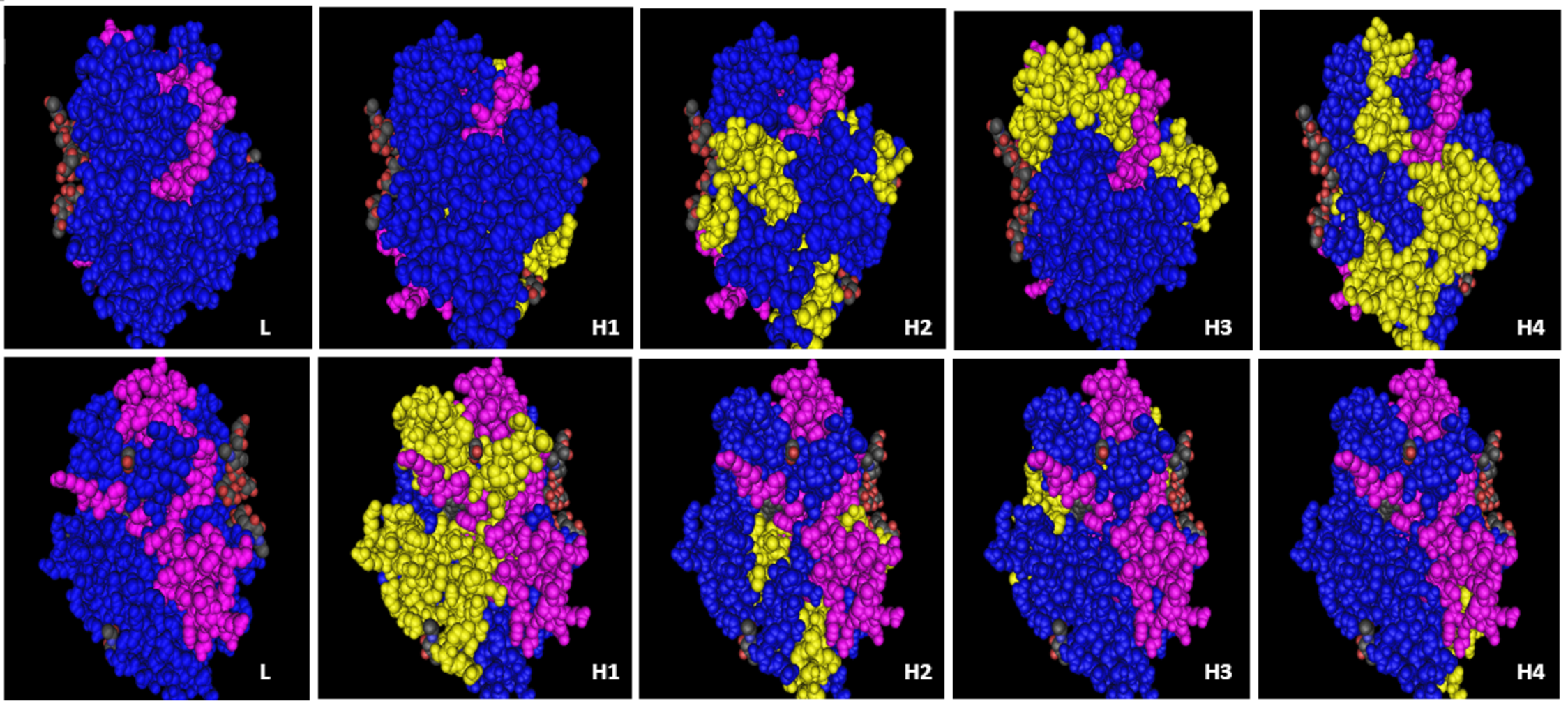

Supplement: Additional file 3: Figure S3 — Location of L, H1, H2, H3, and H4 fragments on MPO. Description: L, H1, H2, H3, and H4 fragments were marked in different colors in one heavy-light protomer unit (MMDB ID 75307) as follows: L by purple; H1, H2, H3, and H4 by yellow. The second row was viewed from the opposite side of the first row. [file ar4386-S3.tiff]
